# A Direct Interaction between Leucine-rich Repeat Kinase 2 and Specific β-Tubulin Isoforms Regulates Tubulin Acetylation*

**DOI:** 10.1074/jbc.M113.507913

**Published:** 2013-11-25

**Authors:** Bernard M. H. Law, Victoria A. Spain, Veronica H. L. Leinster, Ruth Chia, Alexandra Beilina, Hyun J. Cho, Jean-Marc Taymans, Mary K. Urban, Rosa M. Sancho, Marian Blanca Ramírez, Saskia Biskup, Veerle Baekelandt, Huaibin Cai, Mark R. Cookson, Daniel C. Berwick, Kirsten Harvey

**Affiliations:** From the ‡ Department of Pharmacology, UCL School of Pharmacy, University College London 29-39 Brunswick Square, London WC1N 1AX, United Kingdom,; the § Cell Biology and Gene Expression Unit, Laboratory of Neurogenetics and; the ¶ Transgenics Section, Laboratory of Neurogenetics, NIA, National Institutes of Health, Bethesda, MD 20892,; the ‖ Department of Neurosciences, Laboratory for Neurobiology and Gene Therapy, and Leuven Research Institute for Neuroscience & Disease (LIND), Katholieke Universiteit Leuven, 3000 Leuven, Belgium, and; the ** Hertie Institute for Clinical Brain Research and German Center for Neurodegenerative Diseases, 72076 Tübingen, Germany

**Keywords:** Lrrk2, Microtubules, Molecular Genetics, Parkinson Disease, Tubulin, GTPase Mutation, RocCOR, Cytoskeletal Dynamics, Growth Cone, Tubulin Acetylation

## Abstract

Mutations in *LRRK2*, encoding the multifunctional protein leucine-rich repeat kinase 2 (LRRK2), are a common cause of Parkinson disease. LRRK2 has been suggested to influence the cytoskeleton as LRRK2 mutants reduce neurite outgrowth and cause an accumulation of hyperphosphorylated Tau. This might cause alterations in the dynamic instability of microtubules suggested to contribute to the pathogenesis of Parkinson disease. Here, we describe a direct interaction between LRRK2 and β-tubulin. This interaction is conferred by the LRRK2 Roc domain and is disrupted by the familial R1441G mutation and artificial Roc domain mutations that mimic autophosphorylation. LRRK2 selectively interacts with three β-tubulin isoforms: TUBB, TUBB4, and TUBB6, one of which (TUBB4) is mutated in the movement disorder dystonia type 4 (DYT4). Binding specificity is determined by lysine 362 and alanine 364 of β-tubulin. Molecular modeling was used to map the interaction surface to the luminal face of microtubule protofibrils in close proximity to the lysine 40 acetylation site in α-tubulin. This location is predicted to be poorly accessible within mature stabilized microtubules, but exposed in dynamic microtubule populations. Consistent with this finding, endogenous LRRK2 displays a preferential localization to dynamic microtubules within growth cones, rather than adjacent axonal microtubule bundles. This interaction is functionally relevant to microtubule dynamics, as mouse embryonic fibroblasts derived from LRRK2 knock-out mice display increased microtubule acetylation. Taken together, our data shed light on the nature of the LRRK2-tubulin interaction, and indicate that alterations in microtubule stability caused by changes in LRRK2 might contribute to the pathogenesis of Parkinson disease.

## Teaser

**Background:** Mutations in the gene encoding leucine-rich repeat kinase 2 (LRRK2) cause Parkinson disease.

**Results:** LRRK2 binds directly to three β-tubulin isoforms at the luminal face of microtubules and suppresses α-tubulin acetylation. Interaction is weakened by the R1441G LRRK2 GTPase domain mutant.

**Conclusion:** LRRK2 modulates microtubule stability.

**Significance:** Deregulation of microtubule-dependent processes likely contribute to neurodegeneration in Parkinson disease.

## Introduction

Mutations in *LRRK2*, encoding leucine-rich repeat kinase 2 (LRRK2)^6^ are a common cause of inherited Parkinson disease (PD) (1, 2). Because *LRRK2* mutation carriers present symptoms and brain pathology very similar to idiopathic PD (1–4), understanding the biological role of LRRK2 could help to uncover new therapeutic strategies for both inherited and sporadic cases. LRRK2 belongs to the ROCO family of proteins, which are characterized by the unique combination of a Roc (Ras of complex proteins) domain with intrinsic GTPase activity and a COR (C-terminal of Roc) domain. The combination of a GTPase domain and a kinase domain suggests a complex role for LRRK2 in cell signaling (5). Additional protein-protein interaction domains, such as leucine-rich repeat (LRR) and WD40 propeller motifs suggest that LRRK2 has multiple protein interactors that potentially localize LRRK2 to different subcellular compartments (1, 2, 4, 5). Although many sequence variants have been reported in *LRRK2*, dominant mutations clearly segregating with PD are only found in the RocCOR tandem domain or the kinase domain (1, 2, 4, 5). LRRK2 kinase and GTPase activity are clearly important for the cytotoxicity and neurite changes observed with LRRK2 mutants in cellular models (6–12). However, the precise mechanisms by which LRRK2 mediates these events remain elusive.

One newly emerging theme is the interaction between LRRK2 and the cytoskeleton. For example, LRRK2 has been shown to interact with microtubules (MTs) (13–23) and influence MAPK and Wnt signaling pathways (19, 24–29). Increased phosphorylation of the MT-associated protein (MAP) Tau, which has been implicated in the pathogenesis of PD in recent genome-wide screens (30, 31), is seen in several animal models expressing LRRK2 mutants (28, 32, 33). Therefore *LRRK2* mutations might influence MT dynamics, which would be predicted to play a role in synaptic and axonal degeneration as observed in postmortem brains of PD patients (34).

Here, we demonstrate a specific and direct interaction between LRRK2 and three β-tubulin isoforms that is mediated by the LRRK2 Roc domain and β-tubulin C termini. We demonstrate that this interaction is dependent on guanidine nucleotide binding and modulated by Roc domain autophosphorylation and disrupted by the pathogenic *LRRK2* mutation R1441G. We also show that lysine 362 (Lys-362) and alanine 364 (Ala-364) in TUBB and TUBB4 underlie the isoform specificity of the LRRK2-β-tubulin interaction. Molecular modeling indicates that Lys-362 is present on an interaction surface in the lumen of MT filaments close to the lysine 40 (Lys-40) acetylation site in α-tubulin. Corroborating this finding, LRRK2 knock-out mouse embryonic fibroblasts (MEFs) show increased tubulin acetylation. Last, we demonstrate that LRRK2 co-localizes with highly dynamic cytoskeletal structures in dopaminergic cells, and that LRRK2 overexpression and mutation impacts upon the morphology of growth cones. Taken together, these data suggest a role for LRRK2 in the regulation of cytoskeletal dynamics with implications for the pathogenesis of PD.

## EXPERIMENTAL PROCEDURES

### 

#### 

##### Expression Constructs and Site-directed Mutagenesis

β-Tubulin cDNAs were amplified from human whole brain first-strand cDNA (Clontech) using *Pfx* DNA polymerase (Invitrogen) and cloned into the YTH vector pACT2 (Clontech), or the mammalian expression vector pRK5FLAG. Cloning resulted in an in-frame fusion of the LexA DNA binding domain (LexA-BD; vector pDS-BAIT), GAL4 activation domain (GAL-AD; vector pACT2), or FLAG epitope tags to the N termini of all expressed proteins. Vectors encoding the LRRK2 Roc domain, COR domain, and RocCOR tandem domain in the pDS BAIT plasmid (Dualsystems Biotech), and full-length myc-tagged LRRK2 in the pRK5 mammalian expression vector, have been described previously (19). Lentiviral plasmids encoding EGFP-tagged LRRK2 wild-type and G2019S were generated excising the 3×FLAG tag from the pCHMWS-3×FLAG-LRRK2 constructs (35, 36) via restriction with NheI and BamHI and an EGFP sequence (excised from the peGFP-C1 plasmid using NheI and BglII) was ligated into place, yielding the pCHMWS-eGFP-LRRK2 wild-type and G2019S lentiviral vector plasmids. Mutations were generated using the QuikChange site-directed mutagenesis kit (Agilent). All constructs were verified by DNA sequencing.

##### Yeast Two-hybrid Assays

The yeast strain L40 (Invitrogen) was co-transformed with wild-type and mutant pDS LRRK2 RocCOR, Roc, or COR bait constructs together with wild-type or mutant pACT2-β-tubulin prey constructs encoding the C termini of tubulins from Phe-317 to the stop codon. Transformations were plated on selective dropout media lacking leucine, tryptophan, and histidine supplemented with 0.5 mm 3-Amino-1,2,4-triazole (for suppression of “leaky” histidine expression) for nutritional selection, or leucine and tryptophan for transformation controls. After incubation at 30 °C for 3–6 days to allow prototropic colonies to emerge, *LacZ* reporter gene assays were performed as previously described (37).

Quantitative YTH assays were performed by resuspending cell pellets in Z-buffer containing 40 mm β-mercaptoethanol followed by lysis in 0.1% (w/v) SDS and 0.1% (v/v) chloroform. All protein interactions were assayed in 3–4 independent experiments in triplicate. After addition of chloropheno-red-β-d-galactopyranoside the color change was recorded at 540 nm and readings adjusted for turbidity of the yeast suspension at 620 nm. The background signal (bait + empty pACT2 vector) was subtracted from each reading and values were normalized to the wild-type RocCOR response, which was set at 100%.

##### Cell Culture, Co-immunoprecipitation, and Western Blotting of HEK293 Cells

HEK293 cells (ATCC CRL1573) were grown in Dulbecco's modified Eagle's medium (DMEM) supplemented with 10% (v/v) fetal bovine serum, 2 mm glutamine, 100 units/ml of penicillin G, and 100 μg/ml of streptomycin at 37 °C in 95% air, 5% CO_2_. Exponentially growing cells were transfected with constructs encoding epitope-tagged β-tubulin and LRRK2 using Lipofectamine LTX reagent (Invitrogen). HEK293 cells were harvested 48 h post-transfection, and lysed in a solution containing 150 mm NaCl, 50 mm Tris (pH 7.5), 5 mm EDTA (pH 8), 0.25% (v/v) Nonidet P-40/complete protease inhibitor mixture (Roche Applied Science). Following centrifugation (4 °C, 15 min, 16,000 × *g*) 1 ml of cell lysate containing ∼700 μg of protein was added to 40 μl of anti-FLAG M2 affinity gel (Sigma) and incubated overnight at 4 °C on a turning disk. The affinity gel was subjected to centrifugation (4 °C, 100 × *g*, 3 min) followed by two washes in 50 mm NaCl, 50 mm Tris, 0.1% (v/v) Triton X-100, two washes in 150 mm NaCl, PBS, 0.1% (v/v) Triton X-100, and two washes in PBS, 0.1% (v/v) Triton X-100. The FLAG fusion proteins were eluted with 150 ng of 3×FLAG peptide (Sigma) for 30 min at RT. The eluates were analyzed by SDS-PAGE and immunoblotting. Approximately 10 μg of protein was loaded into 4–12% (w/v) BisTris pre-cast gels (Invitrogen). Proteins were transferred to polyvinylidine fluoride (PVDF) membranes (Millipore) and nonspecific binding was blocked with 5% (w/v) skimmed milk in PBS plus 0.1% (v/v) Tween 20 or with 20% (v/v) horse serum in PBS. Anti-myc antibody (Sigma) was used at a 1:2,000 dilution and anti-FLAG antibody (Sigma) was used at a 1:3,000 dilution at 4 °C overnight. For detection, a HRP-conjugated anti-rabbit secondary antibody (Santa Cruz) was used at a 1:2,000 dilution together with the SuperSignal West Pico Chemiluminescent Substrate (Pierce).

##### GST Pull-downs

The human LRRK2 Roc domain (amino acids 1328–1516) was cloned into a pGEX4T.1 vector with an N-terminal GST tag using BamHI and XhoI restriction sites. The recombinant GST and GST-Roc domains were expressed in *Escherichia coli* BL21(DE3) (Novagen). Bacterial cultures were grown at 37 °C until the *A*_600_ reached 0.5 and then induced with isopropyl β-d-thiogalactopyranoside at a final concentration of 0.2 mm at 37 °C for 1.5 h. Cells were harvested and lysed by sonication on ice in lysis buffer containing 50 mm Tris-HCl (pH 7.4), 150 mm NaCl, 0.5 mm EDTA, 1 mm DTT, 0.1%, octyl-β-glucoside, 10% glycerol and protease inhibitors (Roche Applied Science). After centrifugation for 20 min at 12,000 × *g*, the supernatant was incubated with glutathione-Sepharose 4B beads (Amersham Pharmacia Biosciences) overnight at 4 °C. The beads were washed extensively twice with lysis buffer containing 500 mm NaCl, then twice with lysis buffer containing 250 mm NaCl and twice with lysis buffer containing 150 mm NaCl.

HEK293FT cells (Invitrogen) and human brain samples (cortex) were lysed in 20 mm Tris-HCl pH 7.5, 150 mm NaCl, 1 mm EDTA, 1% Triton, 10% glycerol and protease inhibitor mixture (Roche Applied Science). Lysates were precleared by centrifugation for 20 min at 12,000 × *g* before pull-downs. 25 μl of glutathione-Sepharose 4B beads with 2 μg of purified recombinant proteins (GST, GST-Roc) were incubated with 3 mg of total cell or brain lysates for 2 h at 4 °C. After six washes (20 mm Tris-HCl, 150 mm NaCl, 1% Triton X-100, 10% glycerol and protease inhibitors), co-purified protein was eluted by addition of 2× SDS sample buffer and then incubated at 95 °C for 5 min. Proteins were separated on 4–20% SDS-PAGE (Bio-Rad), stained with Coomassie Brilliant Blue (Thermo Scientific), and individual bands were excised for MS/MS identification.

##### Generation, Culture, and Western Blotting of Lrrk2 Knock-out and Wild-type Control MEF Cells

Mouse skin primary fibroblast cells (MEF) were derived from the dorsal skin of postnatal day 0 (P0) wild-type or *Lrrk2* knock-out mouse pups (38). Minced skin was suspended in HEPES-buffered DMEM supplemented with 0.25% trypsin and 0.01% DNase I and incubated at 37 °C for 20 min. The tissues were then transferred to DMEM supplemented with 10% fetal bovine serum and dissociated by repeated trituration. Cells were plated and immortalized by transduction with large T antigen. The resulting cell colonies were isolated and propagated individually to generate LRRK2 wild-type and knock-out stable cell lines. For transfection experiments, MEF cells were grown in 6-cm plates and transfected with 2 μg of pRK5myc or pRK5mycLRRK2 using FuGENE HD (Roche). Cells were harvested 24 h post-transfection in buffer containing 50 mm Tris, pH 7.5, 100 mm NaCl, 1% Triton X-100, 1× complete protease inhibitor mixture (Roche), and 1× Halt phosphatase inhibitor mixture (Pierce). Lysates were clarified by centrifugation at 14,000 × *g* for 10 min at 4 °C, and supernatants were retained for Western blot, performed as above. Membranes were probed with mouse acetylated tubulin antibody (6–11B-1; Sigma), rabbit α/β-tubulin antibody (Pierce), and rabbit LRRK2 antibody (MJFF2; Epitomics) at 1:2,000 dilution. In all cases, membranes were probed first for acetyltubulin, incubated for 30 min in stripping buffer (1.5% (w/v) glycine, 1% Tween 20 and 1% SDS, adjusted to pH 2.2), and then re-probed for total tubulin.

##### Culture and Immunocytochemistry of SH-SY5Y Cells

SH-SY5Y cell lines stably over-expressing EGFP-LRRK2 wild-type or EGFP-LRRK2 G2019S were made by lentiviral transduction as described previously (39, 40). Cells were cultured in DMEM supplemented with 1× non-essential amino acids (Invitrogen), 15% (v/v) FBS, and 1% (v/v) gentamycin. Neuronal differentiation was induced by incubation in DMEM containing 1× non-essential amino acids, 5% FBS, 1% (v/v) gentamycin, and 10 μm retinoic acid, with media replaced every 2 days. Wild-type SH-SY5Y cells were cultured in DMEM/F-12 (1:1 Invitrogen) supplemented with 10% (v/v) FBS and 1% (v/v) penicillin/streptomycin. SH-SY5Y cells were differentiated by treatment with 10 μm retinoic acid and subsequent incubation for 4–7 days in Neurobasal medium supplemented with 2 mm l-glutamine, 1% (v/v) penicillin/streptomycin and B27 supplement (Invitrogen). Cells were fixed using a cytoskeletal fixation method described previously to allow extraction of all proteins not associated with the cytoskeleton (41). Cells were stained with antibodies recognizing LRRK2 (Michael J. Fox Foundation), or acetylated tubulin (6–11B-1; Sigma) and counterstained with phalloidin and DAPI for F-actin and nuclei, respectively.

##### Confocal Microscopy and Image Analysis

Confocal microscopy was performed using a Zeiss LSM 710 META. All images were taken with a ×63 objective. Fluorescence exited by the 488, 543, and 633 nm laser lines of argon and helium/neon lasers was detected separately using only one laser at the time (multitrack function) and a combination of band pass filters (BP 505–530, BP 560–615), long pass (LP 560) filters, and meta function (649–798) dependent on the combination of fluorochromes used.

##### Molecular Modeling

Molecular modeling of the tubulin structure was performed using Chimera (42). Amino acid substitutions were made with the *swapaa* command using the Dunbrack backbone-dependent rotamer library (43) and taking into account the lowest clash score, highest number of H-bonds, and highest rotamer probability. For comparison with the taxol binding site, the structure of α/β-tubulin dimers bound to taxol was used (PDB 1JFF) (44). Because this compound alters the surface conformation adjacent to the LRRK2 binding site, the remaining models used the structure of unbound α/β-tubulin dimers (PDB 1TUB) (45).

##### Statistical Analysis

Growth cone width (Fig. 6*I*; *n* = 10) and filopodia number (Fig. 6*J*; *n* = 10) were analyzed first by two-way analysis of variance (ANOVA) to determine significant effects of cell type, differentiation time course, and the interaction between cell type and differentiation. Statistically significant effects were further investigated by one-way ANOVA. Tubulin acetylation (Fig. 7*E*; *n* = 3) was analyzed by unpaired Student's *t* test with two-tailed distribution. Quantitative yeast two-hybrid assays (Figs. 8, *B* and *C* (*n* = 5), *D* (*n* = 6) and 9, *A–C* (*n* = 4)) were analyzed using one-way ANOVA. In all cases, combined data from a minimum of three independent experiments was used. In all cases *p* values are indicated (*, *p* < 0.05; **, *p* < 0.01; ***, *p* < 0.001). Error bars represent the mean ± S.D.

## RESULTS

### 

#### 

##### The Roc Domain Interacts Directly with the C Terminus of Specific β-Tubulin Isoforms

**FIGURE 1.**
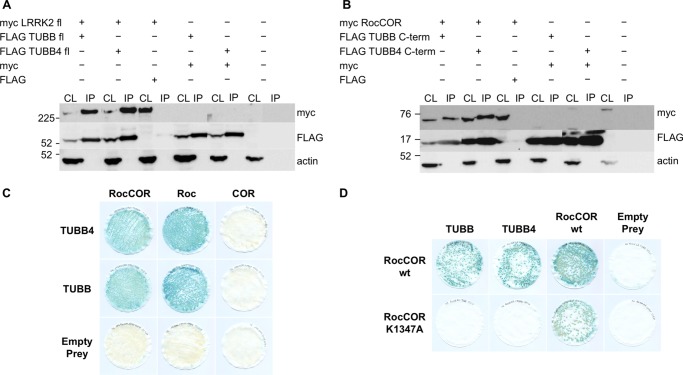
**The LRRK2 Roc GTPase domain binds TUBB and TUBB4.** *A,* full-length myc-tagged LRRK2 interacts with full-length FLAG-tagged TUBB and TUBB4 in co-immunoprecipitation experiments (*CL,* cell lysate; *IP*, = anti-FLAG immunoprecipitates; transfections as indicated). *B,* the LRRK2 RocCOR domain and the C termini of interacting β-tubulin isoforms were sufficient for interaction. *C*, semiquantitative YTH experiments show interaction with TUBB and TUBB4 to require the LRRK2 Roc but not COR domain. *D*, semiquantitative *lacZ* freeze-fracture assays show that the LRRK2 guanine nucleotide-binding mutant K1347A abolishes LRRK2 interactions with TUBB and TUBB4 but not LRRK2 RocCOR dimerization.

A yeast two-hybrid (YTH) screen of a human whole brain cDNA library using a LRRK2 RocCOR domain bait (19) revealed several independent cDNA clones for the C terminus (Phe-317 to Ala-444) of the β-tubulin isoform TUBB4. This interaction was verified in co-immunoprecipitation assays using full-length myc-tagged LRRK2 and full-length FLAG-tagged TUBB4 (Fig. 1*A*), and with the myc-tagged LRRK2 RocCOR domain and the TUBB4 C terminus (Fig. 1*B*). A similar interaction was seen between LRRK2 and another β-tubulin isoform, TUBB (Fig. 1, *A* and *B*).

**FIGURE 2.**
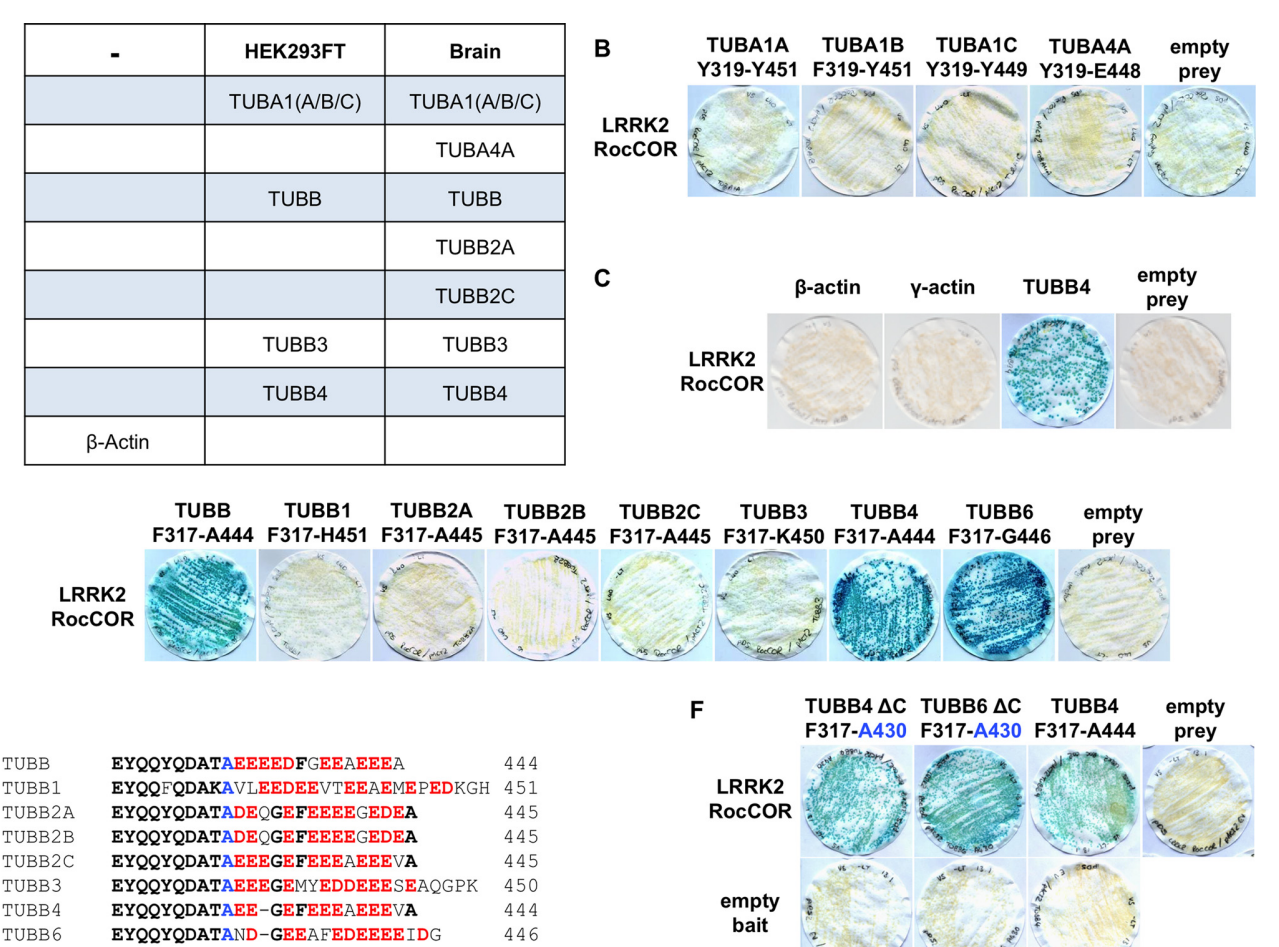
**The direct interaction between LRRK2 and tubulin is specific to TUBB, TUBB4, and TUBB6.** *A,* mass spectrometry of GST-Roc immunoprecipitation from brain and HEK293 cell lysates identified α-tubulin and β-tubulin isoforms but not β-actin as LRRK2 interactors. *B*, YTH assays show that the LRRK2 RocCOR tandem domain does not bind directly to the α-tubulin isoforms TUBA1A, TUBA1B, TUBA1C, and TUBA4A; or *C*, β- or γ-actin. *D,* LRRK2 binds directly to the C termini of TUBB, TUBB4, and TUBB6 but not to TUBB1, TUBB2A-C, or TUBB3. *E,* sequence alignment of the divergent extreme C-terminal tails of all eight β-tubulins. *F,* deletion of these C-terminal amino acid residues does not abolish the interaction between LRRK2 and β-tubulin in YTH assays.

In an independent screening technique, we recovered several tubulin isoforms from HEK293 cell or mouse brain lysates by glutathione pull-downs with a recombinant GST-Roc domain protein (Fig. 2*A*). This refined the interaction to the Roc domain (13). Confirming the localization of interaction, both TUBB and TUBB4 interacted with the isolated LRRK2 Roc domain in YTH assays but neither β-tubulin bound the adjacent COR domain (Fig. 1*C*). Furthermore, interference with the Roc domain function by the introduction of a guanine nucleotide non-binding mutation (K1347A) prevented association with either β-tubulin isoform, although self-interaction of the RocCOR tandem domain was retained (Fig. 1*D*).

The above results suggest that in brain both α- and β-tubulin isoforms can co-precipitate with LRRK2, but because tubulin forms α-/β-heterodimers, either group of proteins could directly interact with LRRK2. To resolve this, we used YTH assays with α-tubulins that were precipitated with recombinant GST-Roc (TUBA1A, TUBA1B, TUBA1C, and TUBA4A; Fig. 2*A*). None of the α-tubulins studied were found to bind the LRRK2 RocCOR tandem domain (Fig. 2*B*), suggesting direct interaction is specific for β-tubulin isoforms. The LRRK2 RocCOR domain also failed to interact with β- or γ-actin (Fig. 2*C*). Next, we tested all eight human β-tubulin isoforms in YTH assays. Besides TUBB and TUBB4, only one other β-tubulin was found to interact, TUBB6 (Fig. 2*D*). Because the extreme C termini are the most diverse part of the β-tubulin isoforms (Fig. 2*E*) we tested whether these regions might mediate the interactions. We found that the region from Phe-317 to the C terminus of TUBB, TUBB4, and TUBB6 was sufficient for interaction with the LRRK2 RocCOR tandem domain (Fig. 2*D*). However, the extreme C terminus was not required for binding. C-terminal deletion constructs (Phe-317 to Ala-430) did not abolish interaction but increased protein-protein interaction strength between β-tubulins and LRRK2 in comparison to β-tubulins with intact C termini (Fig. 2*F*).

**FIGURE 3.**
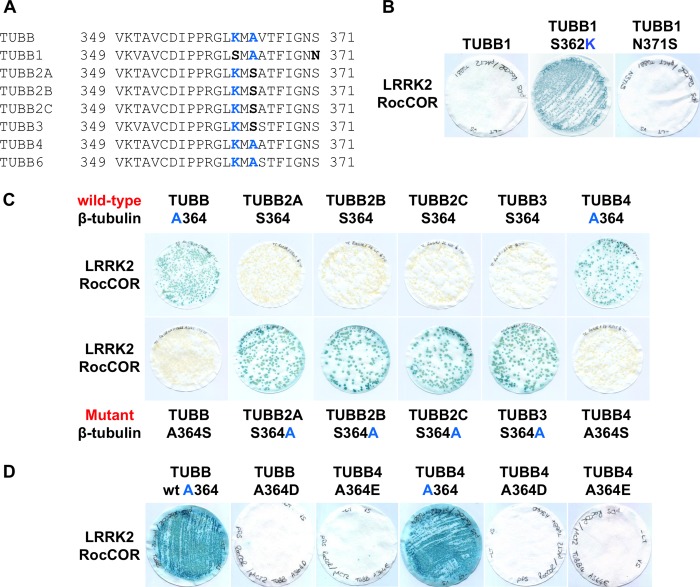
**The LRRK2 β-tubulin interaction requires lysine 362 and alanine 364 of β-tubulin.** *A,* alignment of β-tubulin isoforms reveals further sequence divergence between residues 349 and 371. Note the presence of Lys-362 and Ala-364 in isoforms found to bind LRRK2 (TUBB, TUBB4, and TUBB6). *B,* mutation S362K allows TUBB1 to bind LRRK2. *C,* mutation A364S in TUBB and TUBB4 abolishes the interaction of these proteins with LRRK2. By contrast, the reciprocal S364A mutation in TUBB2A, TUBB2B, TUBB2C, and TUBB3 allows these β-tubulins to interact with LRRK2. *D,* interaction of TUBB and TUBB4 with the LRRK2 RocCOR tandem domain is also abolished by phosphomimetic mutations at residue 364.

Having narrowed the LRRK2 interaction site on β-tubulin to a region between Phe-317 and Ala-430, we further compared the amino acid sequences in this region (Fig. 3*A*). Intriguingly, only three residues were conserved in the three interacting β-tubulins, but at least one of these residues was missing from the non-binding β-tubulin isoforms: Lys-362, Ala-364, and Ser-371. Of the non-binding β-tubulins, TUBB1 has serine at position 362 and an asparagine at position 371, whereas TUBB2A, TUBB2B, TUBB2C, and TUBB3 have a serine residue at position 364. Introduction of a S362K substitution into TUBB1 conferred LRRK2 binding (Fig. 3*B*), confirming a requirement for Lys-362. By contrast, mutation of Asn-371 to the serine present in all other β-tubulins (N371S) had no effect on binding (Fig. 3*B*). S364A mutations in TUBB2A, TUBB2B, TUBB2C, and TUBB3 also permitted the direct association of these proteins with LRRK2 (Fig. 3*C*), whereas the reciprocal A364S substitutions in TUBB and TUBB4 abrogated binding (Fig. 3*C*). We also introduced phosphomimetic mutations into TUBB and TUBB4 (A364D, A364E). These negatively charged amino acids abolished the interaction with LRRK2 in a similar manner to A364S mutations (Fig. 3*D*). Thus, the binding of LRRK2 to β-tubulins requires two near adjacent residues, Lys-362 and Ala-364.

**FIGURE 4.**
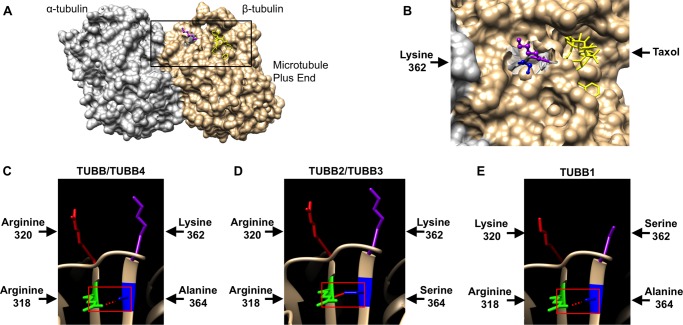
**Lysine 362 and alanine 364 of β-tubulin change the conformation of the LRRK2 binding site.** *A,* modeling shows the LRRK2 and taxol binding sites are in close proximity at the luminal surface of MTs. *B,* magnification of the area indicated in *A. C-E*, modeling of the structural influence of Lys-362 and Ala-364 *versus* Ser-362 and Ser-364 on the accessibility of the LRRK2 binding site. *C,* Ala-364 in TUBB and TUBB4 is unlikely to form hydrogen bonds with arginine 318 allowing for good accessibility of Lys-362 at the MT surface. *D,* by contrast, Ser-364 is predicted to form a hydrogen bond with arginine 318 restricting Lys-362 conformation. *E,* the shorter side chain of Ser-362 in comparison to Lys-362 at the MT surface is predicted to be unable to coordinate LRRK2 binding. *A* and *B* are derived from the crystal structure of bovine α/β-tubulin dimers bound to taxol; *C–E* are derived from the crystal structure of unbound porcine α/β-tubulin dimers.

Because high-resolution crystal structures of α-/β-tubulin heterodimers are available, we utilized a molecular modeling approach to characterize the putative LRRK2 interaction surface on β-tubulin. Lys-362 is exposed at the protein surface, at a location close to the β-tubulin binding site for the MT-stabilizing compound taxol (Fig. 4, *A* and *B*). Both Lys-362 and the taxol binding site are located on the luminal surface of mature MT filaments. Further analysis revealed that the large positively charged side chain of Lys-362 projects away from the rest of the molecule (Fig. 4*B*). This observation is consistent with a key role for this amino acid in docking the LRRK2-tubulin interaction, and in agreement with the failure of TUBB1 to bind LRRK2 (Figs. 2*D* and 3*B*).

Surprisingly, the second amino acid required for LRRK2 binding, Ala-364, is not exposed, residing just below Lys-362 at the top of an internal β-sheet (Fig. 4*C*). To determine whether the presence of serine *versus* alanine at position 364 might alter the external topology, hydrogen bond prediction was performed (Fig. 4, *C–E*). Ala-364 appears unlikely to interact with any neighboring residues (Fig. 4, *C* and *E*). By contrast, Ser-364 allows hydrogen bonding between the hydroxyl side chain of this residue and the peptide backbone of the opposite strand of the β-sheet, at arginine 318 (Fig. 4*D*). This hydrogen bond would be expected to increase stability at the top of the β-sheet, holding the protein loops immediately above in a more rigid conformation. This, in turn, would likely affect the accessibility of Lys-362. Thus we predict that the presence of serine or alanine at position 364 governs LRRK2 binding by modulating β-tubulin surface topology and altering the position of Lys-362.

**FIGURE 5.**
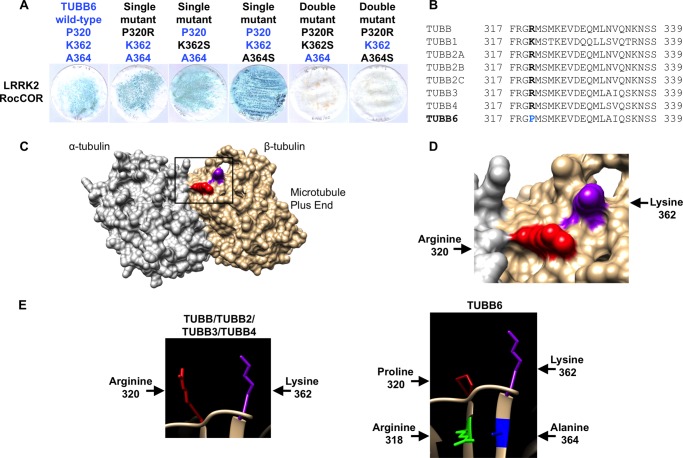
**Mutations to TUBB6 confirm the influence of arginine *versus* proline at residue 320 on interaction with LRRK2.** *A,* the TUBB6 single mutants P320R, K362S, or A364S do not abolish the LRRK2 interaction with tubulin. Double mutants P320R/K362S or P320R/A364S in TUBB6 abolish the interaction with LRRK2. *B,* alignment of β-tubulin isoforms reveals sequence divergence between TUBB6 and all other β-tubulins at residue 320. *C,* modeling shows Arg-320 in β-tubulin in close proximity to the LRRK2 binding residue Lys-362. *D,* magnification of the area indicated in *C. E,* modeling of the structural influence of Arg-320 *versus* Pro-320 on the accessibility of the LRRK2 binding site.

Surprisingly, mutation of the key residues Lys-362 or Ala-364 to serine did not abolish interaction of LRRK2 with TUBB6 (Fig. 5*A*). This suggested the presence of additional residue(s) governing interaction in this β-tubulin. Because molecular modeling suggested the protein loop immediately above Arg-318 might influence interaction with LRRK2 (Fig. 4, *C–E*) we focused on differences between TUBB6 and other β-tubulins in this region. Intriguingly, all β-tubulins except TUBB6 contain a large positively charged amino acid at position 320 (lysine in TUBB1, arginine in the remaining β-tubulins; Figs. 4, *C–E*, and 5, *B* and *E*). In TUBB6, this residue is a small, uncharged proline (Fig. 5, *B* and *E*). Importantly, amino acids at this position are in close proximity to Lys-362 at the microtubule surface (Fig. 5, *C* and *D*) and are therefore likely to affect LRRK2 binding. Thus we hypothesized that the presence of Pro-320 in TUBB6 was sufficient to explain the different behavior of this β-tubulin in YTH assays. Similar to individual K362S and A364S mutations, a P320R amino acid substitution had no effect on LRRK2 binding (Fig. 5*A*). However, when P320R was combined with the K362S or A364S mutations, mimicking TUBB1 and TUBB2A/2B/2C/3, respectively, the resultant TUBB6 proteins were unable to bind LRRK2 (Fig. 5*A*). Thus Pro-320 of TUBB6 is permissive of LRRK2 binding. Conversely, arginine at this position is inhibitory, at least when combined with K362S or A364S mutations. Taken together, this data confirms the reported association between LRRK2 and tubulins, and demonstrates for the first time that interaction is direct and specific to three β-tubulin isoforms, TUBB, TUBB4, and TUBB6. Interaction requires the LRRK2 Roc domain and is dependent on Lys-362 and Ala-364 within the β-tubulin C termini. The interaction is blocked in other β-tubulin isoforms by Ser-362 or the combination of Ser-364 and Arg-320, and also by phosphomimetic amino acids at position 364 in TUBB and TUBB4. Importantly, the interaction surface on β-tubulins is predicted to be adjacent to the taxol binding site at the luminal surface of mature MT filaments.

##### The Direct Binding of LRRK2 to β-Tubulin on the Luminal Face of Microtubule Protofibrils Is Predicted to Affect Microtubule Acetylation

**FIGURE 6.**
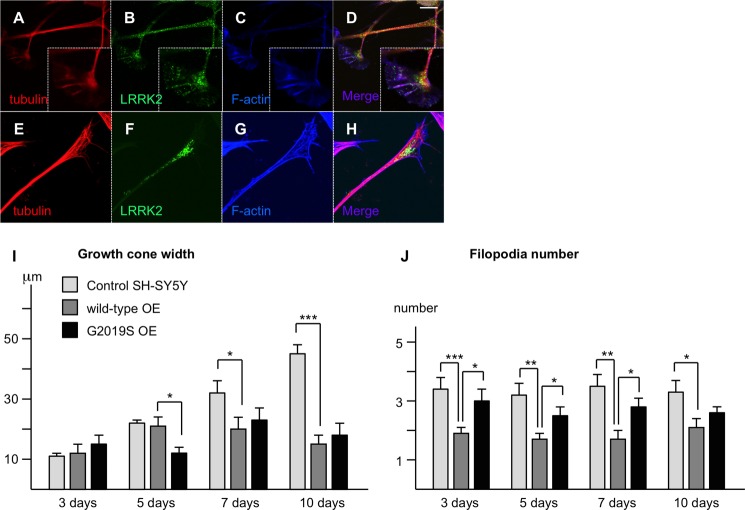
**LRRK2 expression levels and the G2019S mutation affect growth cone dynamics.** *A–H*, endogenous (*A–D*) and stably overexpressed (*E–H*) LRRK2 localizes preferentially to growth cones in SH-SY5Y neurites. Note the decline in expression levels proximal of the growth cones along the neurites. *Scale bar* 10 μm. *I,* the increase in growth cone width during differentiation observed in control cells is abolished in cells over-expressing wild-type or G2019S mutant LRRK2. *J*, the number of filopodia per growth cone is decreased significantly in cells over-expressing wild-type LRRK2 in comparison to control cells. By contrast, the number of filopodia per growth cone is increased significantly in cells over-expressing G2019S mutant LRRK2 in comparison to wild-type over-expressing cells. *, *p* < 0.05; **, *p* < 0.01; ***, *p* < 0.001; *n* = 10.

Molecular modeling predicts that the LRRK2 binding site on β-tubulin is likely to be poorly accessible to a large protein such as LRRK2. This suggests that LRRK2-β-tubulin binding is more likely to occur among dynamic pools of MTs. Dynamic MTs are not acetylated, leading to weaker interprotofibril interactions (46), and a more open, flexible conformation. The dynamic instability of MTs is crucial for growth cone function and LRRK2 has previously been reported to localize to growth cones (19). Here, we examined the association between LRRK2 and growth cones in SH-SY5Y cells during differentiation (Fig. 6). We compared LRRK2 localization in cell lines stably over-expressing EGFP-tagged wild-type LRRK2 (Fig. 6, *E–H*) with normal controls (Fig. 6, *A–D*). Using an established fixation procedure designed to allow detection of cytoskeletal structures (41), confocal microscopic analysis confirmed the presence of LRRK2 in growth cones. Endogenous LRRK2 and EGFP-LRRK2 displayed similar patterns of localization, and were detectable throughout the growth cone and axon shaft, co-localizing with MTs (Fig. 6, *A–H*). LRRK2 was predominantly localized within the central zone (C-zone) of the growth cone, the contrast between this and the relative lack of LRRK2 at adjacent stabilized axonal MTs should be noted (Fig. 6, *B* and *F*). Thus LRRK2 appears to preferentially associate with dynamic MTs, consistent with the notion that a direct interaction site for LRRK2 is more accessible in this MT population.

We also investigated growth cone parameters in SH-SY5Y cells over-expressing wild-type or G2019S LRRK2 to a similar extend and control cells during differentiation. Variables measured included growth cone width (Fig. 6*I*), length, volume, and number of filopodia (Fig. 6*J*). Overall most parameters varied widely, and initial differences were often diminished over time, or did not reach statistical significance. Nonetheless, two-way analysis of variance of growth cone width revealed significant effects of differentiation, cell type, and the interaction between these variables (*p* < 0.001 in each case). Further investigation by one-way ANOVA indicated that the effect of differentiation was entirely restricted to growth cones of control cells, which widened significantly throughout differentiation (Fig. 6*I*; *p* < 0.001). By contrast, increased growth cone width was not observed in cells over-expressing LRRK2 wild-type or the G2019S mutant (*p* = 0.193 and *p* = 0.256, respectively). As a result, growth cones were significantly smaller in over-expressing cells than controls at later time points. Thus, LRRK2 over-expression had a pronounced inhibitory effect on growth cone width expansion that appears to be independent of the G2019S mutation.

By contrast, over-expression of wild-type and G2019S LRRK2 had markedly different effects on the number of filopodia per growth cone (Fig. 6*J*). Differentiation did not alter filopodia number (two-way ANOVA: *p* = 0.378), nonetheless, a highly significant effect of cell type was observed (*p* < 0.001). In comparison to control cells, LRRK2 wild-type over-expression decreased the average number of filopodia per growth cone, with significant differences measured at all time points. Contrastingly, cells over-expressing the G2019S mutant consistently displayed more filopodia than the LRRK2 wild-type over-expressing cell line (Fig. 6*J*). Overall, the over-expression of LRRK2 wild-type and G2019S mutant has clear effects on growth cone parameters, suggesting that LRRK2 has important impacts on neurite outgrowth and synapse formation.

**FIGURE 7.**
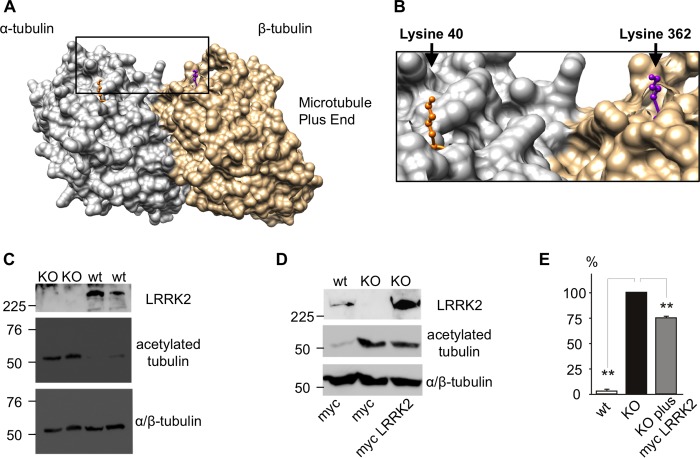
**LRRK2 expressions correlates inversely with tubulin acetylation.** *A,* modeling shows the LRRK2 binding site in close proximity to the Lys-40 acetylation site in α-tubulin at the luminal surface of MTs. *B,* magnification of the area indicated in Fig. 6*A. C,* acetylation of Lys-40 in α-tubulin is massively increased in LRRK2 knock-out MEFs. *D* and *E,* the increase in acetylation in LRRK2 knock-out MEFs can be rescued by over-expression of human LRRK2. *, *p* < 0.05; **, *p* < 0.01; ***, *p* < 0.001; *n* = 3.

MT acetylation/deacetylation occurs on the luminal face of α-tubulin at Lys-40 (47, 48). Because this residue is proximal to the LRRK2 binding site on TUBB, TUBB4, and TUBB6 (Fig. 7, *A* and *B*), we investigated whether a functional connection between LRRK2 expression and MT acetylation exists. To this end, the level of acetyl-α-tubulin in LRRK2 knock-out MEFs was determined by Western blotting. Compared with a wild-type control cell line, LRRK2 knock-out MEFs display a striking increase in tubulin acetylation (Fig. 7, *C–E*). Importantly, transient over-expression of human wild-type LRRK2 in a subset of knock-out MEFs rescued this phenotype (Fig. 6, *D* and *E*). Note that a 25% decrease in acetyl-α-tubulin compares very favorably with a maximal transfection efficiency of ∼40% observed under these conditions.^7^ These data are consistent with a model where direct LRRK2 interaction with TUBB, TUBB4, and TUBB6 on the luminal face of MTs is able to modulate tubulin acetylation.

##### The R1441G LRRK2 Mutation and Mutations Mimicking LRRK2 Roc Domain Autophosphorylation Modulate Binding to β-Tubulin

**FIGURE 8.**
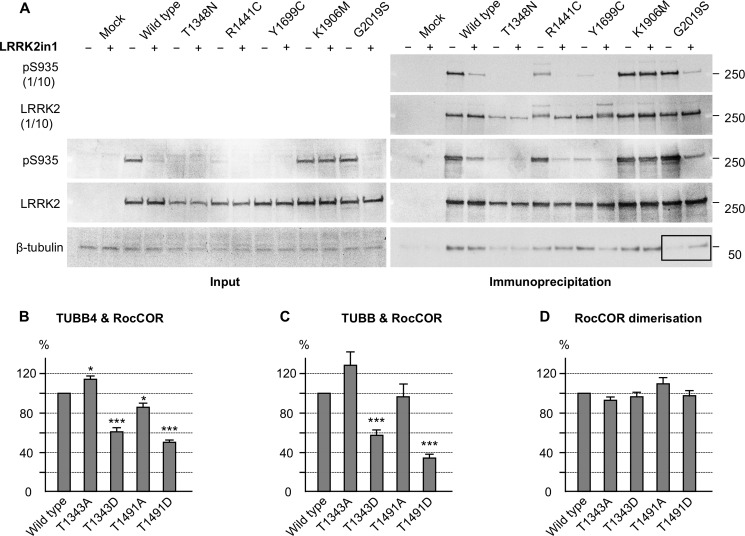
**LRRK2 Roc domain autophosphorylation interferes with the LRRK2-tubulin interaction.** *A,* Western blot showing a decrease of the LRRK2-β-tubulin interaction in co-immunoprecipitation experiments with the G2019S mutant in comparison with wild-type LRRK2 (*black box*). This decrease was rescued by LRRK2 kinase inhibition with LRRK2in1. *B–D*, quantitative YTH assays show that the introduction of phosphomimetic mutations at Roc domain autophosphorylation sites decreases the interaction with TUBB4 (*B*) and TUBB (*C*) but has no effect on RocCOR dimerization (*D*). Introduction of an alanine at the autophosphorylation sites has less and more diverse effects on the interactions shown. *, *p* < 0.05; **, *p* < 0.01; ***, *p* < 0.001; *n* = 5–6.

Using co-immunoprecipitation, we investigated the effect of a number of mutations in full-length LRRK2 on tubulin binding in HEK293 cells (Fig. 8*A*). Neither familial *LRRK2* nor artificial mutations in the Roc, COR, or kinase domains of LRRK2 (T1348N, R1441C, Y1699C, G2019S, K1906M), had statistically significant effects on LRRK2 interaction with β-tubulin (Fig. 8*A*). Similar results were obtained when co-precipitating α-tubulin (data not shown). LRRK2 kinase inhibitors have been reported previously to modulate the association of LRRK2 with MTs, albeit with contradictory results (16, 18). In our hands, the LRRK2-in-1 kinase inhibitor (49) failed to impact on LRRK2-β-tubulin interactions (Fig. 8*A*). Nonetheless, we observed a decrease in the interaction between β-tubulin and the LRRK2 G2019S mutant with increased kinase activity that was rescued by pharmacological inhibition of LRRK2 kinase activity (Fig. 8*A, black box*).

We hypothesized that co-immunoprecipitation might lack the sensitivity to detect changes in LRRK2-tubulin interaction strength. Thus, the effect of LRRK2 mutants on the interaction with TUBB and TUBB4 was investigated using quantitative YTH assays (Q-YTH; Figs. 8, *B–D,* and 9, *A–C*). Because LRRK2 autophosphorylation within the Roc domain has been reported (50–52), we investigated the effect of phosphomimetic amino acid substitutions at two Roc domain autophosphorylation sites on LRRK2-β-tubulin interactions, as a proxy for elevated kinase activity. Both mutants, T1343D and T1491D, weakened the interactions with TUBB/TUBB4 by ∼40–65% (Fig. 8, *B* and *C*). Mutation of the same residues to alanine led to only a small reduction, no reduction, or an increase of the interaction strength. Importantly, none of these mutations influenced LRRK2 RocCOR dimerization strength, demonstrating that these mutations did not influence the Q-YTH results non-specifically (Fig. 8*D*).

**FIGURE 9.**
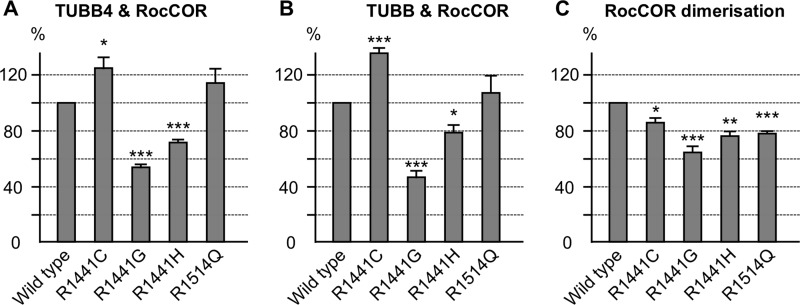
**Familial *LRRK2* mutations affect the LRRK2 β-tubulin interaction.** *A* and *B*, quantitative YTH assays show that the introduction of familial *LRRK2* mutations can increase (R1441C) as well as decrease (R1441G/H) the interaction with TUBB4 (*A*) and TUBB (*B*), whereas the R1514Q amino acid change, which does not segregate with PD, has no statistically significant effect. *C,* all amino acid changes decrease the RocCOR dimerization strength. *, *p* < 0.05; **, *p* < 0.01; ***, *p* < 0.001; *n* = 4.

We next investigated the effect on interaction with TUBB and TUBB4 of PD-associated mutations in the LRRK2 Roc domain. Interestingly, a *LRRK2* mutation with proven pathogenicity, R1441G, clearly reduced LRRK2-tubulin interactions by ∼50% (Fig. 9, *A* and *B*), as did R1441H albeit to a lesser extent (∼25%). By contrast, R1441C appeared to increase LRRK2-tubulin interactions by ∼25%, whereas a LRRK2 variant not segregating with PD (R1514Q) (53) showed no significant effect on the interaction between LRRK2 and TUBB/TUBB4 (Fig. 8, *A* and *B*). The R1441C, R1441G, R1441H and R1514Q amino acid changes all decreased RocCOR dimerization strength between 60 and 80% (Fig. 9*C*) indicating that the results obtained for the LRRK2-tubulin interactions are not the result of nonspecific behaviors of the RocCOR baits in the Q-YTH assay. These data are consistent with an inhibitory effect of selected Arg-1441 mutations and LRRK2 kinase activity on direct MT binding. Because G2019S is known to increase LRRK2 kinase activity, these observations strongly suggest that this mutation, like R1441G or R1441H, will result in weakened LRRK2 binding to β-tubulin.

## DISCUSSION

This study defines the interaction between LRRK2 and the cytoskeleton, demonstrating that the LRRK2 Roc domain binds specifically to three neuronally expressed β-tubulin isoforms, TUBB, TUBB4, and TUBB6, but not the other common isoforms, TUBB1, TUBB2A/B/C, and TUBB3. This suggests that LRRK2 distribution along MTs is determined by the tubulin composition of MTs, in particular by the types of β-tubulin present. This specificity might also account for differences in LRRK2 association with MTs in different brain regions or cell types. We mapped the LRRK2-β-tubulin interaction surface to a site centered around Lys-362, which is located on the same surface of α/β-tubulin heterodimers as two sites important for the modulation of MT stability: the binding site for the MT-stabilizing drug taxol on β-tubulin (Fig. 4, *A* and *B*) and the MT acetylation site on α-tubulin at Lys-40 (Fig. 7, *A* and *B*). These observations suggested that LRRK2 binding to MTs could modulate MT stability. Consistent with this view, LRRK2 knock-out MEF cells show a marked increase in MT acetylation at the key α-tubulin residue Lys-40 (Fig. 7, *C–E*), indicative of greater MT stability. Whether this represents increased acetylation or decreased deacetylation (or a combination of both mechanisms) cannot yet be determined. Nonetheless, it is interesting to speculate as to the mechanism by which the LRRK2 protein might decrease Lys-40 acetylation. In mammals, the major enzyme catalyzing tubulin acetylation is the α-tubulin Lys-40 acetyltransferase (also known as MEC-17) (47, 54), whereas deacetylation is carried out by two enzymes, histone deacetylase 6 (HDAC6) (48, 55, 56) and sirtuin2 (SIRT2) (57). In principle LRRK2 could alter the activity of any one of these enzymes, for example, via protein-protein interaction, or alternatively via phosphorylation. However, given the large size of LRRK2 (see following paragraph), we would suggest that the most likely explanation is that LRRK2 simply prevents α-tubulin Lys-40 acetyltransferase from accessing Lys-40, thereby keeping α-tubulin in a non-acetylated state. Clearly, these ideas require further testing. Nonetheless, we conclude that LRRK2-tubulin interactions affect MT acetylation and thereby promote MT destabilization.

We also note that Lys-362 has been reported to be a site of ubiquitination in two proteomic studies (TUBB2B, TUBB3, and TUBB6 (58); TUBB, TUBB2C, TUBB3, TUBB4, and TUBB6 (59)). Ubiquitinated Lys-362 would be predicted to block LRRK2 association with MTs. Therefore, a dynamic regulation of LRRK2-β-tubulin interactions and by extension MT acetylation via Lys-362 ubiquitination in TUBB, TUBB4, and TUBB6 is plausible.

Our hypothesis is that LRRK2-β-tubulin binding interferes with tubulin acetylation and occurs predominantly in dynamic pools of MTs with a more open, flexible conformation (46). Consistent with this idea, we found a preferential localization of LRRK2 to MTs within growth cones, rather than adjacent stable MTs in axons (Fig. 6, *B* and *F*). Growth cone function relies on the dynamic instability of MTs and dynamic MTs display weaker interprotofibril associations. This creates greater luminal space, allowing for the binding of a large molecule such as LRRK2. LRRK2-β-tubulin interactions are likely to be of functional relevance to growth cone biology, because over-expression of LRRK2 during differentiation results in a reduction in growth cone width (Fig. 6*I*) and number of filopodia per growth cone (Fig. 6*J*). Nonetheless, the location of the LRRK2 binding site on β-tubulins does raise the question of whether this protein might interact with MTs within mature, stable MT tracts, such as those in axons. It should be noted that the presence of protein complexes within MT filaments is well supported in the literature. Indeed, the MAP Tau has been demonstrated to partially reside inside this compartment (60). However, whereas LRRK2 seems able to access the lumen of dynamic MT structures at growth cones, we would suggest that it is less likely that LRRK2 can access the β-tubulin binding site along the entire length of neurites within the MT lumen. An electron microscopic study of cross-sectioned MT filaments in *Drosophila* revealed an average internal area of 244 nm^2^, indicating that MTs with an internal diameter greater than 19 nm are extremely rare in nature (46). Assuming a tubulin width of ∼6.5 nm (46), this internal diameter can be considered equivalent to three tubulin monomers at most. The crystal structure of LRRK2 has not been resolved and thus the physical size of this protein is unknown. Nonetheless, at 2527 amino acids in length, LRRK2 is over five times the size of a tubulin monomer. Thus it seems likely that LRRK2 is too large to enter this compartment. In conclusion, LRRK2 appears to bind directly to the lumen of MTs interfering with tubulin acetylation *in vivo*. This binding is likely restricted to locations where MT protofibrils are held in an “open” confirmation.

We also demonstrated that the Roc-domain R1441G (and the less frequent familial R1441H) mutant with proven pathogenicity disrupted LRRK2-β-tubulin interactions, whereas the R1441C variant increased LRRK2-β-tubulin interactions. A trend toward an increased LRRK2-β-tubulin interaction for the R1441C mutant was previously reported (13). By contrast, the non-segregating R1514Q variant (53), showed no influence on LRRK2-β-tubulin interactions (Fig. 9). This observation suggests that altered LRRK2-β-tubulin interactions are likely to occur in patients with these *LRRK2* mutations. This suggests that the interaction between LRRK2 and MTs requires fine regulation, and that both decreased and increased interaction strength could affect the dynamic instability of MTs. Both increased and decreased MT stability are detrimental, well illustrated by the effect of anticancer chemotherapeutic medication increasing (*e.g.* taxol) or decreasing (*e.g.* vincristine) MT stability. Interestingly, interaction strength was also markedly weakened by two mutations (T1343D, T1491D) mimicking LRRK2 autophosphorylation within the LRRK2 Roc domain (Fig. 8, *B* and *C*). These phosphomimetic mutants serve as a proxy for increased LRRK2 kinase activity, which cannot be tested directly in the RocCOR tandem domain constructs used in our YTH assays. Importantly, elevated kinase activity has been a consistent observation for the most common G2019S *LRRK2* mutation, and thus these data suggest the G2019S mutation will also lead to decreased luminal MT binding. Thus, our data indicate that three pathogenic *LRRK2* mutations, G2019S, R1441G, and R1441H, are likely to cause reduced LRRK2-β-tubulin interactions. Interestingly, effects of over-expression of wild-type LRRK2, the R1441G and G2019S mutants on the expression and phosphorylation of the MAP Tau were shown previously (32, 33), whereas in R1441C knock-in mice no effect on Tau expression or phosphorylation was observed (61). This might be an effect of R1441G over-expression in comparison to endogenous expression levels of the R1441C mutant in knock-in mice, but might also correspond to different effects of these mutants on the interaction between LRRK2 and MTs as suggested in this study (Fig. 8, *A* and *B*).

Intuitively, one would expect processes relevant to PD pathogenesis to be modulated similarly by all LRRK2 mutants segregating with the disease. However, different effects of LRRK2 mutants have been described previously in numerous cellular and biochemical assays. These include opposite effects on measurements of LRRK2 kinase activity and protein-protein interaction strength, for example, with 14-3-3 and DVL proteins (8, 19, 62). As such, it is perhaps more likely that PD-relevant processes perturbed by LRRK2 mutants are under fine regulation, with “too much” and “too little” equally able to elicit neurodegeneration. It is thus fascinating that MT overstabilization and destabilization will have detrimental effects on numerous MT-dependent processes. Taken together therefore, our data shed light on the cells biological role of the LRRK2-tubulin interaction, and indicate that alterations in microtubule stability caused by changes in LRRK2 could contribute to the pathogenesis of PD.

Mutations in human tubulin genes have been found in a number of genetic disorders. These include lissencephaly/SBH (*TUBA1A*) (63, 64), asymmetric polymicrogyria (*TUBB2B*) (65), and the ocular motility disorder CFEOM3 (*TUBB3*) (66–68). Most intriguingly, however, an R2G amino acid substitution in TUBB4 has recently been reported as causing the rare movement disorder, dystonia type 4 (also known as whispering dysphonia) (69, 70). This mutation was found to elicit a decrease in TUBB4 expression (69, 70). Because our work has established LRRK2 as a direct interactor of TUBB4, one might expect dystonia type 4 patients to display decreased LRRK2 localization to MTs. In light of the likely decrease in LRRK2 MT binding in R1441G and G2019S carriers, this is an intriguing possibility, suggesting that LRRK2 might be connected to the pathogenesis of an additional movement disorder. This also suggests the possibility that mutations in human tubulin genes might be a genetic risk factor for PD, and we would contend that a genetic analysis of selected tubulin genes (particularly *TUBB*, *TUBB4*, and *TUBB6*) in PD patients is warranted. In any case, it is fascinating that proteins encoded by two genes linked to familial movement disorders should interact directly, suggesting the possibility of a common pathomechanism, and highlighting the potential importance of the LRRK2-β-tubulin interaction.

## Footnotes

7 D. C. Berwick and K. Harvey, unpublished data.

## Footnotes

6 The abbreviations used are:

LRRK2: leucine-rich repeat kinase 2
PD: Parkinson disease
ANOVA: analysis of variance
YTH: yeast two-hybrid
Q-YTH: quantitative YTH
MT: microtubule
MEF: mouse embryonic fibroblast
EGFP: enhanced green fluorescent protein
BisTris: 2-[bis(2-hydroxyethyl)amino]-2-(hydroxymethyl)propane-1,3-diol.
